# Supplementary figures and images for: Regulatory Mechanism of Proanthocyanidins in Grape Peels Using vvi-miR828a and Its Target Gene VvMYBPA1
Source: Plants (Basel). 2024 Jun 18;13(12):1688. doi: 10.3390/plants13121688 (PMC11207263; doi:10.3390/plants13121688)

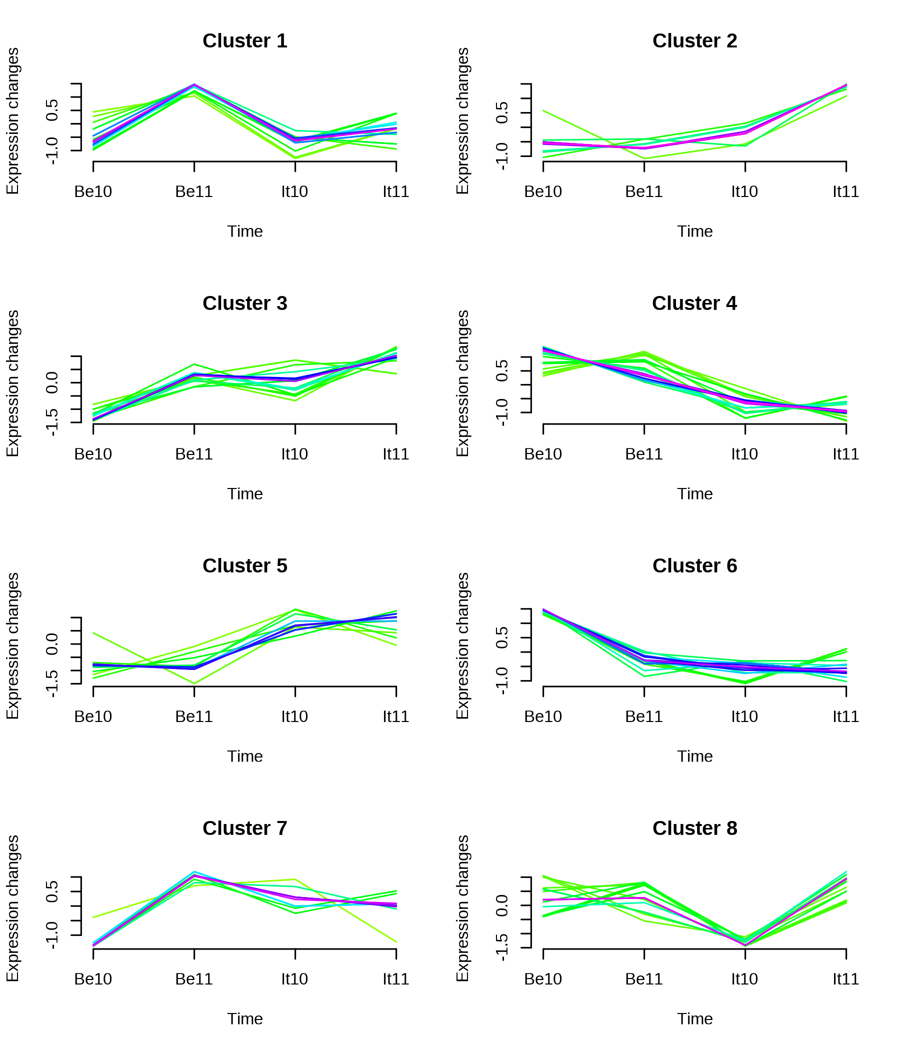

Supplement: Supplementary file 1 [file plants-13-01688-s001.zip › Figure S1.tif]

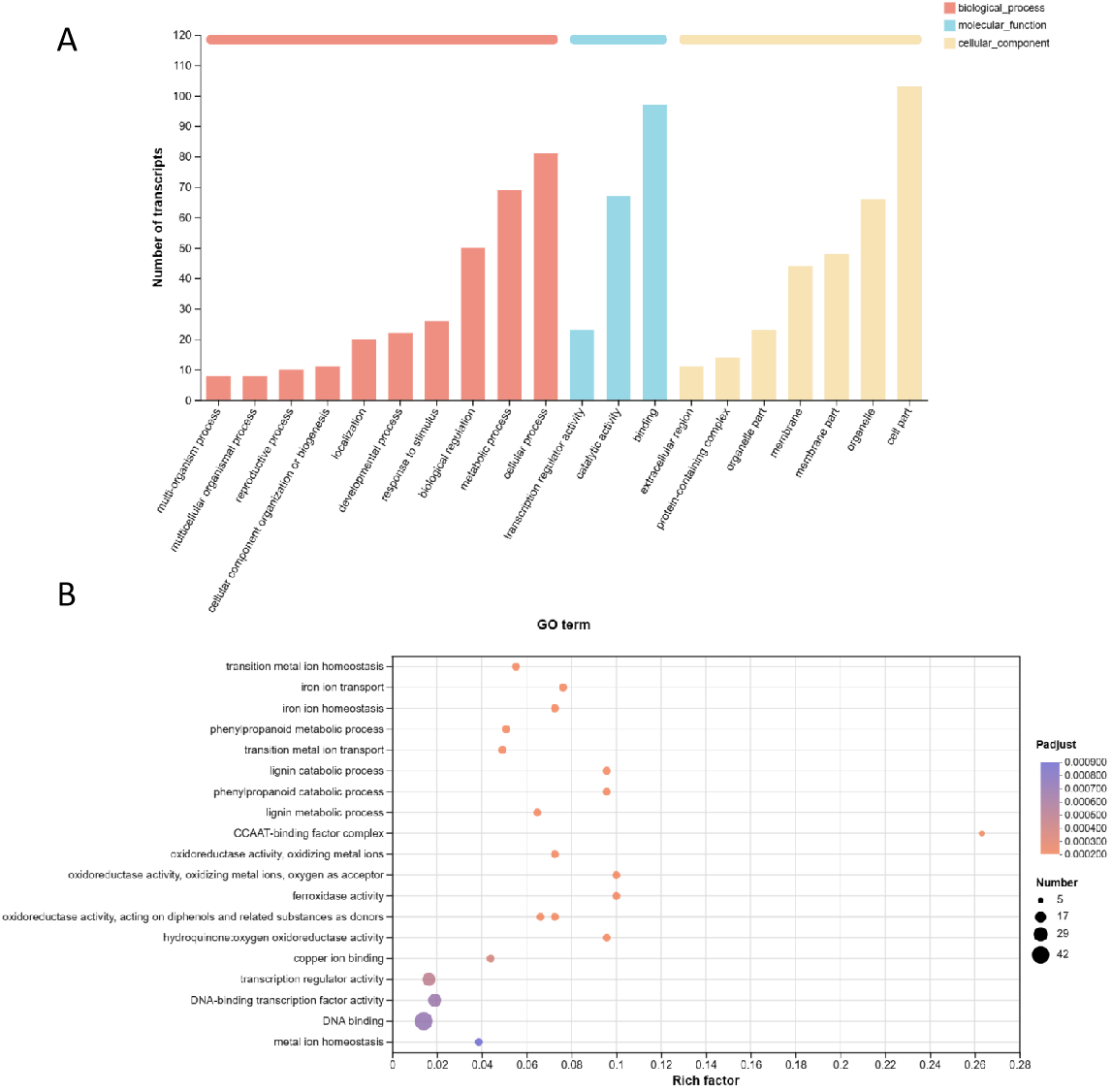

Supplement: Supplementary file 1 [file plants-13-01688-s001.zip › Figure S2.tif]

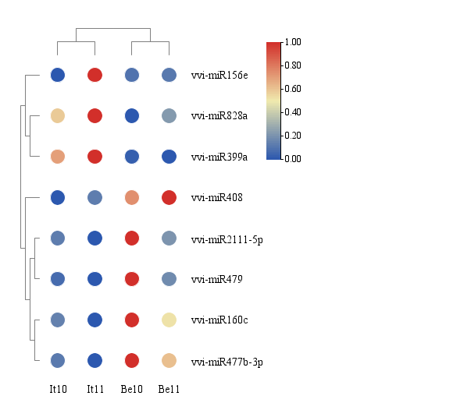

Supplement: Supplementary file 1 [file plants-13-01688-s001.zip › Figure S3.tif]
